# A dual role of transient receptor potential melastatin 2 channel in cytotoxicity induced by silica nanoparticles

**DOI:** 10.1038/srep18171

**Published:** 2015-12-11

**Authors:** Peilin Yu, Jin Li, Jialin Jiang, Zunquan Zhao, Zhaoyuan Hui, Jun Zhang, Yifan Zheng, Daishun Ling, Lie Wang, Lin-Hua Jiang, Jianhong Luo, Xinqiang Zhu, Wei Yang

**Affiliations:** 1 Department of Toxicology, School of Public Health, Zhejiang University, Hangzhou, Zhejiang 310058, P. R. China; 2 Department of Neurobiology, Key Laboratory of Medical Neurobiology of the Ministry of Health of China, Zhejiang University School of Medicine, Hangzhou, Zhejiang 310058, P. R. China; 3 Institute of Immunology, Zhejiang University School of Medicine, Hangzhou, Zhejiang 310058, P. R. China; 4 College of Pharmaceutical Sciences, Zhejiang University, Hangzhou, Zhejiang 310058, P. R. China; 5 School of Biomedical Sciences, Faculty of Biological Sciences, University of Leeds, Leeds LS2 9JT, United Kingdom; 6 Department of Physiology and Neurobiology and Key Laboratory of Brain Research of Henan Province, Xinxiang Medical University, Xinxiang 453003, P. R. China

## Abstract

Silica nanoparticles (NPs) have remarkable applications. However, accumulating evidence suggests NPs can cause cellular toxicity by inducing ROS production and increasing intracellular Ca^2+^ ([Ca^2+^]_i_), but the underlying molecular mechanism is largely unknown. Transient receptor potential melastatin 2 (TRPM2) channel is known to be a cellular redox potential sensor that provides an important pathway for increasing the [Ca^2+^]_i_ under oxidative stress. In this study, we examined the role of TRPM2 channel in silica NPs-induced oxidative stress and cell death. By quantitation of cell viability, ROS production, [Ca^2+^]_i_, and protein identification, we showed that TRPM2 channel is required for ROS production and Ca^2+^ increase induced by silica NPs through regulating NADPH oxidase activity in HEK293 cells. Strikingly, HEK293 cells expressing low levels of TRPM2 were more susceptible to silica NPs than those expressing high levels of TRPM2. Macrophages from young mice showed significantly lower TRPM2 expression than those from senescent mice and had significantly lower viability after silica NPs exposure than those from senescent ones. Taken together, these findings demonstrate for the first time that TRPM2 channel acts as an oxidative stress sensor that plays a dual role in silica NPs-induced cytotoxicity by differentially regulating the NADPH oxidase activity and ROS generation.

With the development of nanotechnology over the past 10 years, nanomaterials have been extensively used in industry, food, cosmetics, and drug delivery. Studies have focused on their unique physical and chemical characteristics as well as the potential adverse effects arising from these properties. Among the many kinds of these nanomaterials, silica (SiO_2_) nanoparticles (NPs) have great promise for applications in biosensors, DNA delivery, sunscreen lotions, and cancer therapy^1^,^2^. Despite many efforts to understand the potential hazardous effects of silica NPs, including oxidative stress and pro-inflammatory responses in rodents and RAW264.7 cells^3^, autophagy and endothelial dysfunction *via* the PI3K/Akt/mTOR signaling pathway^4^, acute inflammation and hepatocyte necrosis through neutrophil-mediated liver injury^5^, as well as apoptosis and changes of the cell cycle in HaCaT cells^6^, there is still a lack of clear understanding of the cytotoxic effects of silica NPs on components of the immune system that provide the first line of defense, such as neutrophils, monocytes, and macrophages. Thus, it is necessary to investigate the responses of macrophages and other immune cells to NPs in order to understand their systemic effects. Besides, although numerous studies have proposed that reactive oxygen species (ROS) play a key role in the cytotoxicity induced by silica NPs, information about ROS generation and modulation remains limited. In addition, cytotoxicity induced by silica NPs has also been associated with disruption of intracellular Ca^2+^ homeostasis^7^,^8^, but the underlying mechanisms are largely unknown.

Recent studies have shown that the TRPM2 channel forms a Ca^2+^-permeable cationic channel activated by intracellular ADP-ribose (ADPR), H_2_O_2_, and ROS^9^,^10^,^11^,^12^, indicating that it serves as a cellular redox potential sensor. In particular, this channel provides an important pathway for oxidative stress-induced increases in the intracellular Ca^2+^ concentrations ([Ca^2+^]_i_) in many cell types^13^. Therefore, we proposed that the TRPM2 channel plays a role in mediating the cytotoxicity induced by silica NPs. There has been no report of any ion channel associated with nanomaterial-induced oxidative stress, which also led us to investigate whether the TRPM2 channel is involved in such a process.

It is well-known that TRPM2 channels are expressed in many cell types in the immune system, such as dendritic cells, polymorphonuclear leukocytes, monocytes, and macrophages, and play a crucial role in producing pro-inflammatory cytokines in response to oxidative stress and inflammatory stimuli^14^. However, the expression pattern of many ion channels and receptors, including TRPM2 channels, is very complicated, making it difficult to evaluate their roles. In addition, a previous study indicated that the functional TRPM2 expression level in pyramidal neurons changes during different development stages in mice^15^, suggesting that the number of TRPM2 channels expressed on the cell surface is important. Here, in order to clarify the role of the TRPM2 channels in silica NPs-induced cytotoxicity, we investigated their detailed function in the human embryonic kidney (HEK293) cell line, since this cell line has few endogenous ion channels and is recognized as the most suitable recombinant system in investigating the functions of ion channels.

In this study, HEK293 cells were used as negative controls, and HEK293 cells showing low (TRPM2-LE) or high (TRPM2-HE) expression level of TRPM2 channels were used to mimic different stages of development. Bone marrow-derived macrophages (BMDMs) from mice of different ages were also used to evaluate the effect of silica NPs on the survival of immune cells. The cell viability, ROS generation, and [Ca^2+^]_i_ were compared between controls and silica NPs-exposed groups. Different inhibitors were applied to identify the functions of TRPM2 channels and the source of ROS generation. Expression of the subunits of NADPH oxidase, which is responsible for silica NPs-induced ROS generation, was also examined.

## Results

### Silica NPs induce differential cytotoxicity in TRPM2-expressing HEK293 cells

**Figure 1:**
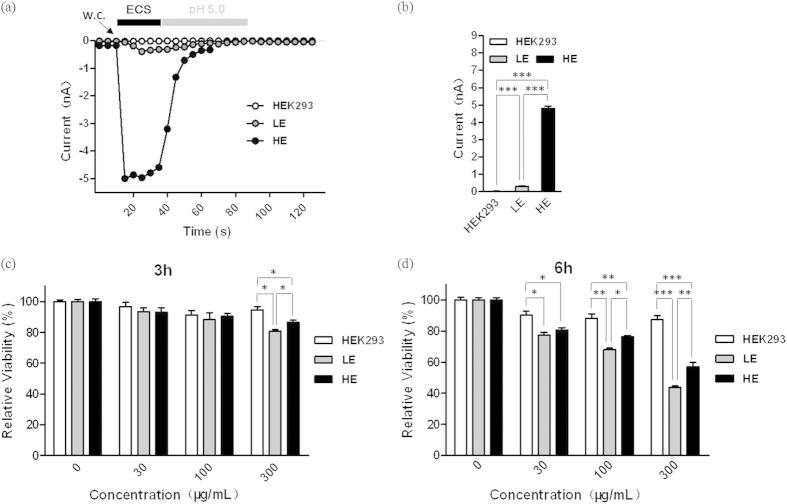
Silica NPs-induced cytotoxicity in HEK293 cells with different TRPM2 expression levels. (**a**) Representative ADPR-induced currents from blank, TRPM2-LE, and TRPM2-HE HEK293 cells; w.c. denotes the start of whole-cell configuration. (**b**) Mean peak currents from recordings as in (**a**), n ≥ 8 cells for each case; ***P < 0.005. (**c**,**d**) Summary of the relative viability of HEK293 cells after exposed to the indicated concentrations of silica NPs for 3 h (**c**) and 6 h (**d**). Untreated cells were used as negative controls. The data are from 10 independent experiments. *P < 0.05, **P < 0.01, ***P < 0.005.

To determine whether the TRPM2 channels are involved in the cytotoxicity induced by silica NPs, we used patch clamp recording of ADPR-induced currents to confirm the different expression levels of TRPM2 channels in three HEK293 cell types (blank, TRPM2-LE and TRPM2-HE). There was no detectable ADPR-induced current in blank HEK293 cells (Fig. 1(a,b)), showing no functional expression of TRPM2 channels in these cells. The average amplitude of ADPR-induced currents in the TRPM2-HE cells was >10 times higher than that in the TRPM2-LE cells (Fig. 1(a,b)). Next, the silica NPs-induced cytotoxicity in these cells was tested by MTT assays. To fully evaluate their effects, three concentrations (30, 100, and 300 μg/mL) of silica NPs and two exposure time (3 h and 6 h) were used. The results showed that silica NPs at all concentrations had no influence on the viability of blank HEK293 cells, but decreased the cell viability in a dose- and time-dependent manner in both TRPM2-LE and TRPM2-HE cells (Fig. 1(c,d)), suggesting that the TRPM2 channels are required for silica NPs-induced cytotoxicity. Surprisingly, we noted that the TRPM2-HE cells had a higher viability than TRPM2-LE cells, and this was dependent on the concentration of silica NPs and the exposure time. These findings indicate that TRPM2 channels mediate differential outcomes in silica NPs-induced cytotoxicity, and the higher expression of TRPM2 channels confer greater cell viability.

### ROS generation plays a critical role in silica NPs-induced cytotoxicity

**Figure 2:**
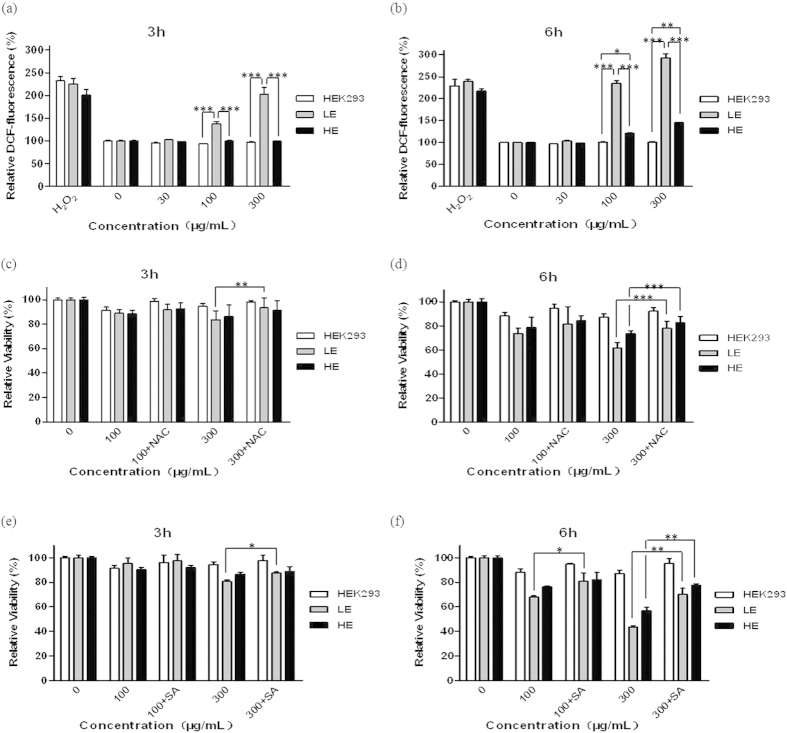
Silica NPs-induced ROS production and cell death in HEK293 cells with different TRPM2 expression levels. (**a**,**b**) Summary of intracellular ROS production in HEK293 cells after exposed to the indicated concentrations of silica NPs for 3 h (**a**) and 6 h (**b**). Untreated cells were used as negative controls and cells treated with 1 mM H_2_O_2_ as positive controls. (**c**–**f**) Summary of the relative viability of HEK293 cells after exposed to the indicated concentrations of silica NPs for 3 h (**c**) and 6 h (**d**) alone, or together with NAC (10 mM; (**c**,**d**)) or SA (5 mM; (**e**,**f** )). Untreated cells were used as negative controls. The data are from 8 independent experiments, *P < 0.05, **P < 0.01, ***P < 0.005.

Previous studies have reported that silica NPs induce ROS generation in several cell types^2^,^16^,^17^,^18^. To clarify whether the cell death caused by silica NPs in our study was due to excessive ROS generation, the fluorescent dye DCFH-DA was used to assess the ROS production. Under our experimental conditions, silica NPs increased slightly ROS production in blank HEK293 cells, but the increase was not statistically significant as compared with untreated cells (Fig. 2(a,b)). Exposure to 100 and 300 μg/mL silica NPs for 3 h led to robust increases in ROS production in the TRPM2-LE cells, but there was no significant increase in ROS production in the TRPM2-HE cells, showing that ROS production induced by silica NPs mainly occurred in cells with the low TRPM2 expression level. Moreover, the ROS production in the TRPM2-LE cells induced by silica NPs exposure for 6 h was higher than that induced for 3 h. Although the ROS production in the TRPM2-HE cells was much lower than in the TRPM2-LE cells after silica NPs exposure for 6 h, it was still significantly higher (*P* < 0.05) than that in blank HEK293 cells, suggesting that the ROS production induced by silica NPs was determined by different TRPM2 expression level. In other words, the ROS production in HEK293 cells expressing a low level of the TRPM2 channels resulted in cell death, but high TRPM2 expression led to inhibition of the ROS production and cell death induced by silica NPs. To determine these results, we examined the effects of pretreatment with N-acetyl-L-cysteine (NAC), a widely-used ROS scavenger, on silica NPs-induced death in these cells. Pre-incubation with 10 mM NAC prevented the death of TRPM2-LE cells induced by exposure to 300 μg/mL silica NPs for 3 h (Fig. 2(c)), indicating that TRPM2 channel-mediated ROS production is essential for the cell death induced by silica NPs. Besides, NAC treatment clearly increased the viability of both TRPM2-LE and TRPM2-HE cells after exposure to 300 μg/mL silica NPs for 6 h (Fig. 2(d)). To further confirm these results, we used another ROS inhibitor, sodium L-ascorbate (SA), which is a direct scavenger of free radicals. The data clearly showed that SA, like NAC, was also effective in preventing the death of both TRPM2-LE and TRPM2-HE cells induced by silica NPs (Fig. 2(e,f)). Taken together, these results show that ROS generation depends on the TRPM2 expression level and plays a critical role in silica NPs-induced cell death.

### TRPM2 bi-directionally regulates NADPH oxidase-mediated ROS generation induced by silica NPs

**Figure 3:**
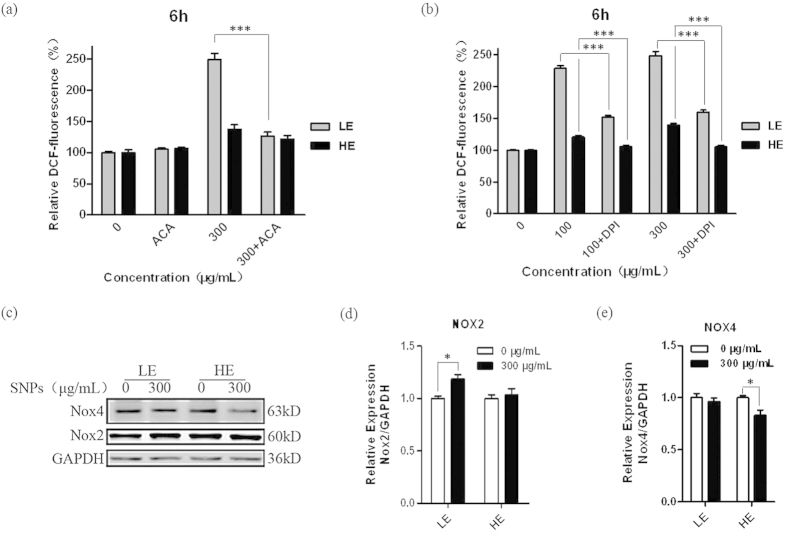
ROS production and change in NADPH oxidase expression in TRPM2-LE and TRPM2-HE cells exposed to silica NPs. (**a**,**b**) Summary of intracellular ROS production in the TRPM2-LE and TRPM2-HE cells after exposed to silica NPs at the indicated concentrations for 6 h alone or together with 20 μM ACA (**a**) and 10 μM DPI (**b**). Untreated cells were used as negative controls. The data were from 5 independent experiments, ***P < 0.005. (**c**–**e**) Representative western blot (**c**) and summary of protein expression of NOX2 (**d**) and NOX4 (**e**) in the TRPM2-LE and TRPM2-HE cells after exposed to 300 μg/mL silica NPs for 6 h. The data in (**d,e**) were from 8 independent experiments. *P < 0.05.

To further clarify the role of TRPM2 channels in regulating the ROS production induced by silica NPs in both TRPM2-LE and TRPM2-HE cells, we used the TRPM2 inhibitor N-(p-amylcinnomoyl) anthralic acid (ACA). The ROS production was measured in control cells and cells treated with ACA and incubated with 300 μg/mL silica NPs for 6 h (Fig. 3(a)). ACA strongly reduced the ROS generation in both types of cells (*P* < 0.001) (Fig. 3(a)), which clearly showed that TRPM2 channels play an important role in the ROS production during exposure to silica NPs.

It is well known that numerous enzyme systems are responsible for producing ROS in mammalian cells, including the NADPH oxidases, xanthine oxidases, NO synthase, and the mitochondrial electron transport chain^19^. Although some studies have reported an increase in the ROS generation in cells exposed to silica NPs, the mechanisms of ROS production remained unknown. Since a recent study reported that TRPM2 channels play a role in the regulation of NADPH oxidase activity^20^, we determined whether NADPH oxidases are involved in TRPM2-mediated ROS generation. Surprisingly, treatment with the widely-used NADPH oxidase inhibitor diphenylenelodonium chloride (DPI) largely abolished the ROS production in both TRPM2-LE and TRPM2-HE cells exposed to silica NPs for 6 h (Fig. 3(b)), indicating that the NADPH oxidases are mainly responsible for the TRPM2-mediated ROS production induced by silica NPs. To further identify the mechanism by which TRPM2 channels regulate NADPH oxidases, the expression levels of the NADPH oxidase subunits NOX1, NOX2, and NOX4 were measured, since they were reported to abundantly express in HEK293 cells^21^. NOX1 expression was not detectable in the HEK293 cells we used (data not shown), but both NOX2 and NOX4 proteins were expressed (Fig. 3(c)). Interestingly, after incubation with 300 μg/mL silica NPs for 6 h, the NOX2 expression in the TRPM2-LE cells was higher than that in the TRPM2-HE cells (Fig. 3(d)), but there was no change in the expression of NOX4 (Fig. 3(e)), suggesting that silica NPs induced ROS production in the TRPM2-LE cells by up-regulating the NOX2 activity. On the contrary, the NOX4 expression was descreased in the TRPM2-HE cells (Fig. 3(e)). So the up-regulated NOX2 expression might be an important factor that produces ROS and mediates cell death in the TRPM2-LE cells, while the inhibition of NOX4 partially protects cells from oxidative stress-induced death in the TRPM2-HE cells. Taken together, these results suggest that the different expression of TRPM2 channels regulate the expression of NOX2 and NOX4, which are involved in silica NPs-induced ROS production.

### TRPM2 expression is associated with the disruption in the Ca^2+^ homeostasis induced by silica NPs

**Figure 4:**
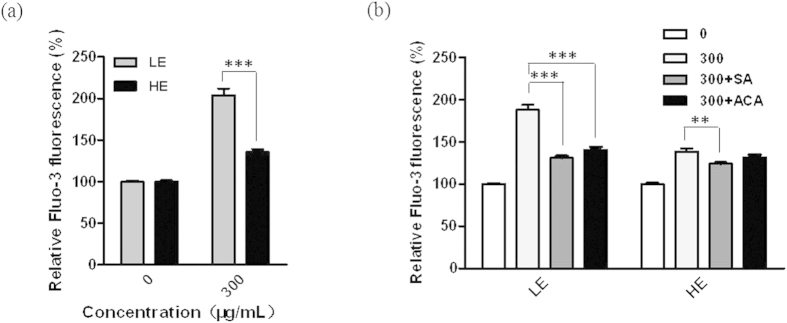
Intracellular calcium concentrations in TRPM2-LE and TRPM2-HE cells exposed to silica NPs. [Ca^2+^]_i_ exposed to silica NPs (300 μg/mL) for 6 h, without (**a**) and with (**b**) SA (5 mM) or ACA (20 μM). Untreated cells were used as negative controls. The data were from 6 independent experiments. **P < 0.01; ***P < 0.005.

To further determine whether TRPM2-mediated ROS generation disrupts the Ca^2+^ homoeostasis after silica NPs exposure, Fluo-3 was used to detect the changes in [Ca^2+^]_i_. Surprisingly, after incubation with 300 μg/mL silica NPs for 6 h, the [Ca^2+^]_i_ in the TRPM2-LE cells was markedly higher (*P* < 0.001) than in the TRPM2-HE cells (Fig. 4(a)), suggesting that silica NPs-induced increase in the [Ca^2+^]_i_ depends on the TRPM2 expression. Moreover, both the ROS scavenger SA and the TRPM2 inhibitor ACA reduced (*P* < 0.001) the increase in the [Ca^2+^]_i_ induced by silica NPs in the TRPM2-LE cells (Fig. 4(b)), which further confirmed that TRPM2-mediated ROS production is responsible for the increase in the [Ca^2+^]_i_. We also noted a much lower Ca^2+^ level in the TRPM2-HE cells than in the TRPM2-LE cells after exposure to silica NPs, which corresponded well with the results for ROS levels under the same conditions (Fig. 2(a,b)), suggesting that ROS production is critical for the Ca^2+^ increase induced by silica NPs. ACA failed to completely abolish the Ca^2+^ increase induced by silica NPs in both the TRPM2-LE and TRPM2-HE cells (Fig. 4(b)), indicating that a TRPM2-independent increase in Ca^2+^ might also be involved in silica NPs-induced cytotoxicity. Taken together, these findings have revealed that ROS production mediated by TRPM2 activation is critical for the increase in the [Ca^2+^]_i_ induced by silica NPs in the TRPM2-LE cells.

### Silica NPs induce differential cytotoxicity in BMDMs from mice of different ages

**Figure 5:**
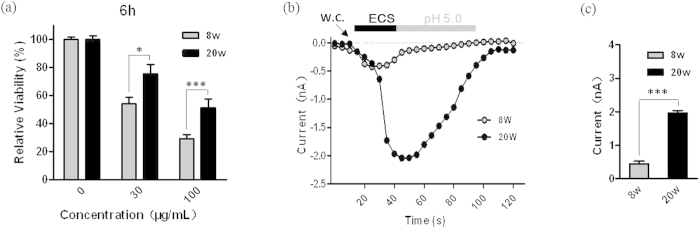
Age-dependent silica NPs-induced cytotoxicity in mouse BMDMs. (**a**) Summary of the relative viability BMDMs from 8 week old (8w) or 20 week old mice (20w) after exposed to 30 and 100 μg/mL silica NPs for 6 h. Untreated cells were used as negative controls. The data were from 5 independent experiments. *P < 0.05; ***P < 0.005. (**b**) Representative ADPR-induced currents in 8w and 20w mouse BMDMs. (**c**) Summary of peak ADPR-induced currents. n ≥ 6 cells in each case. ***P < 0.005.

TRPM2 is highly expressed in cells of the immune system, such as macrophages and monocytes that are mainly responsible for the clearance of exogenous particles. We therefore investigated the effect of silica NPs on the immune cell viability and the role the TRPM2 channels play in this process. Similar to HEK293 cells, we found dose-dependent cytotoxicity of silica NPs in primary cultured BMDMs (Fig. 5(a)). Moreover, the cytotoxicity was age-dependent; cells from the young mice (8 weeks) were less viable than those from the senescent mice (20 weeks) in response to the exposure to both concentrations of NPs (30 μg/ml, *P* < 0.05 and 100 μg/mL, *P* < 0.001) for 6 h (Fig. 5(a)). Based on the above data, we investigated whether the TRPM2 expression differed in macrophages between young and senescent mice. To address this, we determined the functional expression of TRPM2 channels by patch-clamp recording. As expected, ADPR-induced currents in BMDMs from the senescent mice were much higher than those from the young mice (Fig. 5(b,c)), suggesting that the expression of TRPM2 channels is higher on the senescent mouse BMDMs and the differential cytotoxicity of silica NPs in macrophages depends on the TRPM2 expression level. Taken together, our results provide the first evidence that the age-dependent expression of TRPM2 channels in macrophages is critical for the cytotoxicity of silica NPs.

## Discussion

**Figure 6:**
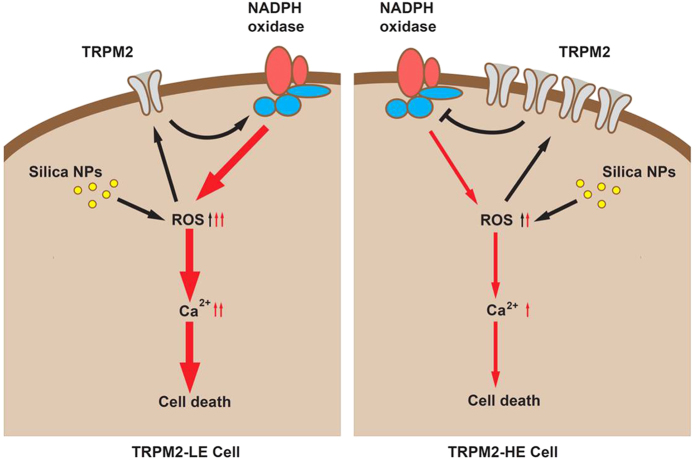
Proposed models for bi-directional regulation by TRPM2 of silica NPs induced cytotoxicity. According to our results, exposure to silica NPs initiates ROS production through TRPM2-independent pathway. In the TRPM2-LE cells (left panel), silica NPs-induced ROS production activates the TRPM2 channels, leading to up-regulation of the NOX2 expression and increased NADPH oxidase activity. This positive feedback results in further ROS generation and increases in the [Ca^2+^]_i_, leading to severe cell death. In contrast, in the TRPM2-HE cells (right panel), silica NPs also increase the ROS production, which also activates the TRPM2 channels in the plasma membrane but inhibit the NADPH oxidase activity by down-regulating the NOX4 expression. This negative feedback reduces the ROS generation and increase in the [Ca^2+^]_i_, and alleviates the cytotoxicity induced by silica NPs.

Numerous studies have been conducted to investigate the health and environmental implications of nanomaterials for humans, some of which focused on the silica NPs examined here. In this study, we have made several novel findings. 1) TRPM2 channels are required for silica NPs-induced cytotoxicity; 2) The expression level of TRPM2 channels is critical in conferring susceptibility to silica NPs-induced cell death (Fig. 1); 3) silica NPs-induced ROS production and increase in the [Ca^2+^]_i_ depend on the expression level of TRPM2 channels that bi-directionally regulates the activity of NADPH oxidase (Figs 2, 3, 4); and 4) although exposure to both 30 and 100 μg/mL silica NPs for 6 h reduced the viability of BMDMs, the viability of BMDMs from the young mice was much lower than in those from senescent mice, and this was correlated with the TRPM2 expression levels (Fig. 5), as seen in HEK293 cells. Taken together, these findings demonstrate that the TRPM2 channel is required for silica NPs-induced ROS production and increase in the [Ca^2+^]_i_, which leads to cell toxicity (Fig. 6). To our knowledge, this is the first report that the TRPM2 channel is critically involved in mediating NPs-induced cell toxicity.

Previous studies suggest that silica NPs-induced cytotoxicity is mainly associated with oxidative stress and increased [Ca^2+^]_i_, but the molecular mechanism still remained elusive. The TRPM2 channel is known as a ROS-activated ion channel that can mediate Ca^2+^ and Zn^2+^ entry into cells^22^,^23^. TRPM2 channels occur in many kinds of cells including macrophages^13^. Although accumulating evidence shows that the TRPM2 channels play a critical role in the immune cell death induced by bacteria, inflammation, and liposomes^24^,^25^,^26^,^27^,^28^, it is still debated whether they play a detrimental or protective role in the immune processes. Given these suggestions, we addressed whether the TRPM2 channels are involved in silica NPs-induced cell toxicity.

First, we found that although without significant difference, exposure to different doses of silica NPs for 3 or 6 h had a little effect on the viability in TRPM2-lacking blank HEK293 cells, which was consistent with the slight increase of ROS in blank HEK293 cells under the same exposure (Fig. 2(a,b)). This suggests that silica NPs probably initiate a small amount of ROS production in blank HEK293 cells through a TRPM2-independent pathway. A recent study reported toxicity and oxidative stress induced by 24 h exposure of HEK293 cells to silica NPs^2^. The disparity between the two studies may be due to the different exposure durations as well as differences in the materials used.

Second, by using HEK293 cells with different TRPM2 expression levels, we presented the first evidence to show that TRPM2 is involved in NPs-induced toxicity. Although not determined directly, it is unlikely that silica NPs directly activate the TRPM2 channels. Despite that the TRPM2 expression level was very low in the TRPM2-LE cells (Fig. 1), our results indicate that the ROS and Ca^2+^ levels were greatly increased after silica NPs exposure, suggesting that TRPM2 is a ROS amplifier under this condition (Figs 2 and 4). Unexpectedly, while the viability of HEK293 cells with high TRPM2 expression level was reduced by silica NPs exposure, these cells were more resistant to silica NPs than the TRPM2-LE cells, manifesting a diminished ROS production and Ca^2+^ increase (Figs 1, 2 and 4). In addition, by using several ROS scavengers (NAC, SA, and DPI), we found that all of these compounds prevented the ROS production induced by silica NPs in the TRPM2-LE cells, indicating that the NADPH oxidase complex is mainly responsible for the ROS generation that leads to cell death (Figs 2 and 3). To further address the mechanism by which TRPM2 regulates the ROS production induced by silica NPs, we performed biochemical assays of NOX2 and NOX4 expression in the TRPM2-LE and TRPM2-HE cells. Our results showed that the NOX2 expression in the TRPM2-LE cells was up-regulated after silica NPs exposure, and this did not occur in the TRPM2-HE cells (Fig. 3). The NOX4 expression was however reduced after silica NPs exposure in the TRPM2-HE cells (Fig. 3). These findings indicated that the different expression of TRPM2 impacts silica NPs-induced cell death by differentially regulating the expression of NADPH oxidases. It would be of interest to investigate in future how the TRPM2 expression level differentially regulates the expression levels of NOX2 and NOX4 induced by silica NPs. In addition, since the few TRPM2 channels expressed in the TRPM2-LE cells were unable to dramatically increase the Ca^2+^ level after silica NPs exposure, one possible explanation is that TRPM2 mediates a positive feedback in the ROS production which elevates the [Ca^2+^]_i_ and induces toxicity. Both ACA and SA prevented the Ca^2+^ increase induced by silica NPs in the TRPM2-LE cells (Fig. 4).

On the other hand, it was surprising to find that the TRPM2-HE cells displayed less toxicity induced by silica NPs than the TRPM2-LE cells, suggesting that high expression level of TRPM2 channels protects cells by mitigating the ROS production and Ca^2+^ increase; this is supported by recent studies^20^,^24^,^29^. A recent study has shown that activation of the TRPM2 channels leads to membrane depolarization and thereby inhibits the membrane potential-sensitive NADPH oxidase activity in macrophages, which limits endotoxin-induced lung inflammation^20^. Together, these findings suggest that the activation of a large number of TRPM2 channels in the TRPM2-HE cells alleviate the toxicity induced by silica NPs and this might result from inhibiting the NADPH oxidase activity, reducing the NOX4 expression or both.

Finally, TRPM2 has been found in many cell types in the immune system, including dendritic cells, polymorphonuclear leukocytes, monocytes, and macrophages^30^,^31^, which are sensitive to many NPs^31^,^32^,^33^. We therefore investigated the role of TRPM2 in silica NPs-induced cell toxicity in primary macrophages. As seen in HEK293 cells, silica NPs induced substantial cytotoxicity in BMDMs from both young and senescent mice (Fig. 5). Importantly, the viability of macrophages from senescent mice was much higher than that from young mice after silica NPs exposure (Fig. 5), suggesting this might be due to the differentTRPM2 expression in these mice as seen in HEK293 cells. Indeed, whole-cell patch-clamp recording demonstrated that functional expression of the TRPM2 channels in macrophages from senescent mice was much higher than those from young mice (Fig. 5). This is the first evidence showing strong changes in the TRPM2 expression during development. Interestingly, a recent study suggests that TiO_2_ particles play different roles in macrophages from mice of different ages^34^, supporting our hypothesis that the cytotoxicity of NPs to macrophages is age-dependent. To our knowledge, this is the first description that TRPM2 channel mediates cytotoxicity induced by silica NPs through regulating the ROS production and increasing the [Ca^2+^]_i_ in primary macrophages in an age-dependent manner.

So far, the role(s) of TRPM2 channels in many physiological and pathological processes remain contentious. For example, some studies have shown that TRPM2 has a protective role in sepsis, cardiovascular injury, *Listeria monocytogenes*-induced liver damage, and endotoxin-induced pulmonary inflammation^27^, while other studies have indicated that TRPM2 plays a detrimental role in such disorders as chemically-induced colitis, ischemia, and diabetes^22^,^28^,^35^. Therefore, the present study not only provides evidence for age-dependent nanotoxicity in the field of toxicology but also offers a possible explanation for the existence of both detrimental and protective functions that may be associated with the TRPM2 expression levels.

## Conclusions

In summary, our experiments provide evidence for the importance of TRPM2 channels in mediating ROS production and cytotoxicity caused by silica NPs. Our results demonstrate that the different expression of TRPM2 is associated with its protective or detrimental role during exposure to silica NPs. A better understanding of the underlying molecular mechanisms by which NPs induce cytotoxicity is a prerequisite for the development of novel and safe NPs.

## Methods

### Characterization and preparation of silica nanoparticles

Silicon dioxide nanopowder (silica NPs, 10–20 nm particle size) was from Sigma (637238; St. Louis, MO, USA). The silica NPs were dissolved in distilled water as stock and dispersed using a sonicator before use (160 W, 20 kHz, 5 min; JY 92-IIN; Scientz, Ningbo, China). The characterization was examined in four different media used in the present study (distilled water; Hanks’ balanced salt solution (HBSS); Dulbecco’s modified Eagle’s medium (DMEM)/F-12 supplemented with 10% fetal bovine serum (FBS); RPMI-1640 supplemented with 10% FBS) using transmission electron microscopy (TEM, Tecnai 10; Philips, The Netherlands), dynamic light scattering (DLS, Nano-S90; Malvern Instruments, UK), and a Zetasizer Nano Series (Malvern Instruments, UK). The diameter of spherical silica NPs, when suspended in above described media, ranged from 40 nm to 60 nm (Fig. S1(a)). Their hydrodynamic size was in the range of 78.5 to 90.9 nm (Fig. S1(b)(c)). The zeta potential determination showed that NPs maintained a negative surface charge. The negative charge value in DMEM and RPMI-1640 culture media was lower than that of H_2_O and HBSS, possibly due to the fact that culture media contained proteins and growth factors (Fig. S1(c)).

### Cell cultures

HEK293 cells were maintained in Dulbecco’s modified Eagle’s medium (DMEM)/F-12 supplemented with 50 units/mL penicillin (15140-122; Gibco, USA), 50 μg/mL streptomycin (15140-122; Gibco, USA) and 10% fetal bovine serum (FBS; 10099-141; Gibco, USA). The TRPM2-LE HEK293 cells were maintained in DMEM/F-12 containing 10% FBS, 80 μg/mL zeocin (ant-zn-1; InvivoGen, USA) and 10 μg/mL blasticidin (ant-bl-1, InvivoGen, USA). For the TRPM2-HE cells, the medium was replaced by FBS containing DMEM/F-12 with 1 μg/mL tetracycline (T3383; Sigma, USA) to induce high expression TRPM2 level. All cells were cultured at 37 °C under a humidified atmosphere containing 5% CO_2_.

Primary cultured bone marrow-derived macrophages (BMDMs) were prepared from C57BL/6 mice. Male mice (8 or 20 weeks old) were maintained under a 12-h light/dark cycle with unlimited access to food and water. Mice were sacrificed to collect the femurs and tibiae. After removing the muscles, the femurs and tibiae were immediately placed in a 30-mm dish (430166; Corning Inc., Corning, NY, USA) containing sterile Hanks’ balanced salt solution (HBSS; 14025-092; Gibco). The bones were flushed with a syringe filled with HBSS to extrude the bone marrow, which was then gently dispersed with a pipette. The cell suspension was centrifuged at 1000 rpm for 5 min. Red blood cells were lysed with ammonium chloride-potassium lysis buffer (150 mM NH_4_Cl, 10 mM KHCO_3_, and 100 mM EDTA) for 5 min. After another centrifugation at 1000 rpm for 5 min, the cell pellet was re-suspended and differentiated in RPMI-1640 (c0003; Gibco) with 10% inactivated FBS, 50 units/mL penicillin, 50 μg/mL streptomycin, and 20 ng/mL macrophage colony-stimulating factor (315-02; PeproTech, USA) for 5 days. Then cells were seeded in 96-well plates (3599; Costar, USA) at 2 × 10^4^ cells/well for exposure to silica NPs.

### Patch-clamp recording

Patch-clamp recordings were made in the whole-cell configuration at room temperature using an Axonpatch 200B amplifier (Molecular Devices, Sunnyvale, CA, USA). Cells were kept in extracellular solution (ECS) containing (in mM): 147 NaCl, 2 KCl, 1 MgCl_2_, 2 CaCl_2_, 10 HEPES, and 13 glucose, pH 7.4. Electrodes had a final resistance of 3–5 MΩ when filled with intracellular solution containing (in mM): 147 NaCl, 0.05 EGTA, 1 MgCl_2_, 10 HEPES, and 0.5 ADPR, pH 7.3. The membrane potential was held at 0 mV. To record ADPR-induced currents, voltage ramps with 500 ms duration from −100 mV to 100 mV were applied every 5 s. The inward currents at −80 mV, denoted by circles, are shown in the figures. To confirm that they were TRPM2 channel-mediated currents, we applied ECS at pH 5.0, which completely blocks TRPM2 channels^36^, at the end of each measurement. Change of the acidic ECS was carried out using an RSC-160 system (Bio-logic Science Instruments, Claix, France). For analysis, the mean of the first three ramps before channel activation was used for leak subtraction of all subsequent current recordings.

### Methylthiazolyldiphenyl-tetrazolium (MTT) assay

Cell viability was measured using the MTT assay. Cells were plated into 96-well plates (3599; Costar) at 4 × 10^4^ cells/well and cultured overnight in full medium for complete adherence. Then the cells were exposed to silica NPs (30, 100, or 300 μg/mL) for 3 or 6 h under normal culture conditions. In experiments on the effects of inhibitors, cells were pretreated with 10 mM N-acetyl-L-cysteine (NAC; A7250; Sigma, USA) or 5 mM sodium L-ascorbate (SA; A7631; Sigma, USA) for 1 h before incubation with NPs. For MTT assays, after NPs exposure, the medium was replaced with regular medium, and the cells were incubated with MTT (0.5 mg/mL; M2128; Sigma) at 37 °C for 4 h. Crystals of formazan, the metabolite of MTT, were dissolved with DMSO and measured at an absorbance of 490 nm using a microplate reader (Tecan Infinite M200, Thermo Fisher Scientific Inc., Waltham, MA, USA). The cell survival rate was calculated from the relative absorbance and expressed as a percentage of control. Each group was composed of five replicate wells.

### Measurement of ROS production

Intracellular ROS production was assessed by 2′, 7′-dichlorofluorescin diacetate (DCFH-DA) staining. DCFH-DA enters cells and is deacetylated to form DCFH which is trapped in the cells and oxidized by intracellular ROS to transform into fluorescent DCF. The fluorescence intensity of DCF was used to indicate the intracellular ROS level. Briefly, cells in 96-well plates (3603; Costar) after NPs incubation were washed 3 times with HBSS and incubated with 10 μM DCFH-DA (C2938; Life Technologies, Waltham, MA, USA) at 37 °C for 30 min. Then 2 washes with HBSS were followed by fluorescence imaging at 520 nm emission induced by 485 nm excitation, determined with a full-wavelength multifunction scanning reader (Varioskan Flash; Thermo Scientific). The intensity of the control well was assumed to be 100% and the data are presented as percentage of control. In the studies with inhibitors, cells were pretreated with 10 μM DPI (D2926; Sigma) or 20 μM N-(p-amylcinnomoyl) anthralic acid (ACA; A8486; Sigma) for 1 h prior to NPs incubation. In each measurement, cells incubated with 1 mM H_2_O_2_ for 30 min served as the positive control and each group had 5 replicate wells. All procedures were performed in the dark.

### Western blotting

Cells were stimulated with 300 μg/mL silica NPs for 6 h. After washing with ice-cold PBS, cells were lysed in ice-cold protein lysis buffer (RIPA buffer, P0013B; Beyotime Institute of Biotechnology, Nantong, China) for 30 min. After centrifuging the lysates at 12 000 rpm at 4 °C for 10 min, the supernatants were collected and stored at −80 °C until use. The protein concentrations were determined using a BCA protein assay kit (P0010; Beyotime Institute of Biotechnology). Thirty to fifty micrograms of protein/lane was diluted in standard SDS sample buffer and subjected to electrophoresis on 12% SDS-polyacrylamide gels. Proteins were then transferred to polyvinylidene difluoride membranes (IPVH00010; Millipore, Billerica, MA, USA), blocked with 5% BSA (A0332, Sangon Biotech, Shanghai, China) in Tris-buffered saline containing 0.05% Tween-20 (TBST) for 2 h at room temperature and incubated with the primary antibody (Nox1, ab55831, Abcam, Cambridge, UK, 1:1000 dilution; Nox2, ab129068, Abcam, 1:1000 dilution; Nox4, ab109225, Abcam, 1:1000 dilution; and GAPDH, LCA03, Auragene Bioscience Corp., Morgan Hill, CA, USA, 1:1000 dilution) overnight at 4 °C. The membranes were then washed with TBST and incubated with the secondary antibody FITC-conjugated anti-mouse IgG (926-32220; Li-Cor Biotechnology, Lincoln, NE, USA, 1:5000 dilution) for 1 h. Protein bands were visualized using an Odyssey Infrared Imaging System (Li-Cor Biosciences). Quantity One software (4.6.2; BioRad) was used for densitometric scanning.

### Assessment of intracellular calcium concentration

Free [Ca^2+^]_i_ was determined using Fluo-3/AM (F1242; Life Technologies, Waltham, MA, USA USA). Cells in 96-well plates (3603; Costar) were exposed to silica NPs for 6 h and incubated with 3.5 μM Fluo-3/AM at 37 °C for 1 h. Then the cells were washed with HBSS and the fluorescence intensity was measured using a full-wavelength multifunction scanning reader (Varioskan Flash; Thermo Scientific) with excitation at 485 nm and emission at 525 nm. The intensity of the control well was set at 100% and the data are presented as percentages of control. For the inhibitor experiments, cells were pretreated with 5 mM SA (A7631; Sigma, USA) for 1 h before incubation with NPs. Each group had 5 replicate wells and all procedures were performed in the dark.

### Data analysis

Data are expressed as mean ± SEM, from at least 5 independent experiments. Statistical analysis was performed using two-way ANOVA with the Bonferroni *post-doc* test where appropriate and one-way ANOVA followed by Dunnett’s *t*-test for comparison between groups, with *P* < 0.05 considered to be statistically significant. Prism 5 software was used for all statistical analyses.

## Additional Information

**How to cite this article**: Yu, P. *et al.* A dual role of transient receptor potential melastatin 2 channel in cytotoxicity induced by silica nanoparticles. *Sci. Rep.* **5**, 18171; doi: 10.1038/srep18171 (2015).

## Supplementary Material

Supplementary Information
