# Supplementary material for: A dual role of transient receptor potential melastatin 2 channel in cytotoxicity induced by silica nanoparticles
Source: Sci Rep. 2015 Dec 11;5:18171. doi: 10.1038/srep18171 (PMC4676061; doi:10.1038/srep18171)
Supplement: Supplementary Information [file srep18171-s1.pdf]

Supplementary information

A dual role of transient receptor potential melastatin 2 channel in cytotoxicity induced by silica nanoparticles

Peilin Yu[1*], Jin Li[1*], Jialin Jiang[1], Zunquan Zhao[1], Zhaoyuan Hui[3], Jun Zhang[1], Yifan Zheng[1], Daishun Ling[4], Lie Wang[3], Lin-Hua Jiang[5, 6], Jianhong Luo[2], Xinqiang Zhu[1#], Wei Yang[2#]

[1]Department of Toxicology, School of Public Health, Zhejiang University, Hangzhou, Zhejiang 310058, P. R. China

[2]Department of Neurobiology, Key Laboratory of Medical Neurobiology of the Ministry of Health of China, Zhejiang University School of Medicine, Hangzhou, Zhejiang 310058, P. R. China

[3]Institute of Immunology, Zhejiang University School of Medicine, Hangzhou, Zhejiang 310058, P. R. China

[4]College of Pharmaceutical Sciences, Zhejiang University, Hangzhou, Zhejiang 310058, P. R. China

[5]School of Biomedical Sciences, Faculty of Biological Sciences, University of Leeds, Leeds LS2 9JT, United Kingdom

[6]Department of Physiology and Neurobiology and Key Laboratory of Brain Research of Henan Province, Xinxiang Medical University, Xinxiang 453003, P. R. China

[*]These authors contributed equally to this work.

[#]Correspondence: Wei Yang, Department of Neurobiology, Key Laboratory of Medical Neurobiology of the Ministry of Health of China, Zhejiang University School of Medicine, Hangzhou, Zhejiang, 310058, P. R. China. Tel: +86 57188208244. E-mail: yangwei@zju.edu.cn ; or Xinqiang Zhu, E-mail: zhuxq@zju.edu.cn.

Supplemental Fig. 1. (Fig. S1.)

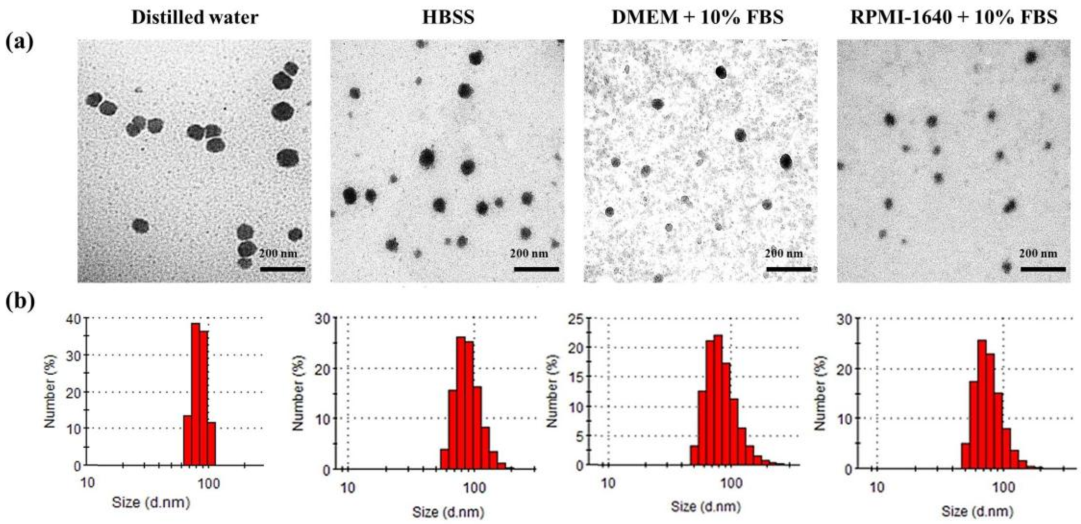

(c)

Medium	Hydrodynamic size（nm）	PDI	Zeta Potential（mv）
Distilled water	85.0 ± 10.8	0.564	-7.93 ± 0.15
HBSS	90.9 ± 21.4	0.266	-8.63 ± 0.53
DMEM + 10% FBS	86.1 ± 27.4	0.325	-5.76 ± 0.73
RPMI-1640 + 10% FBS	78.5 ± 20.4	0.267	-4.21 ± 0.57

Fig. S1. Characterization of silica NPs. TEM images (a), DLS measurements (b) and DLS and zeta potential measurements (c) of silica NPs dispersed 30 μg/mL in four indicated solutions or media used in the study.
